# Galectin-3 and Beclin1/Atg6 Genes In Human Cancers: Using cDNA Tissue Panel, qRT-PCR, and Logistic Regression Model to Identify Cancer Cell Biomarkers

**DOI:** 10.1371/journal.pone.0026150

**Published:** 2011-10-19

**Authors:** Halliday A. Idikio

**Affiliations:** Department of Laboratory Medicine and Pathology, University of Alberta, Edmonton, Alberta, Canada; Queen Elizabeth Hospital, Hong Kong

## Abstract

**Background:**

Cancer biomarkers are sought to support cancer diagnosis, predict cancer patient response to treatment and survival. Identifying reliable biomarkers for predicting cancer treatment response needs understanding of all aspects of cancer cell death and survival. Galectin-3 and Beclin1 are involved in two coordinated pathways of programmed cell death, apoptosis and autophagy and are linked to necroptosis/necrosis. The aim of the study was to quantify galectin-3 and Beclin1 mRNA in human cancer tissue cDNA panels and determine their utility as biomarkers of cancer cell survival.

**Methods and Results:**

A panel of 96 cDNAs from eight (8) different normal and cancer tissue types were used for quantitative real-time polymerase chain reaction (qRT-PCR) using ABI7900HT. Miner2.0, a web-based 4- and 3- parameter logistic regression software was used to derive individual well polymerase chain reaction efficiencies (E) and cycle threshold (Ct) values. Miner software derived formula was used to calculate mRNA levels and then fold changes. The ratios of cancer to normal tissue levels of galectin-3 and Beclin1 were calculated (using the mean for each tissue type). Relative mRNA expressions for galectin-3 were higher than for Beclin1 in all tissue (normal and cancer) types. In cancer tissues, breast, kidney, thyroid and prostate had the highest galectin-3 mRNA levels compared to normal tissues. High levels of Beclin1 mRNA levels were in liver and prostate cancers when compared to normal tissues. Breast, kidney and thyroid cancers had high galectin-3 levels and low Beclin1 levels.

**Conclusion:**

Galectin-3 expression patterns in normal and cancer tissues support its reported roles in human cancer. Beclin1 expression pattern supports its roles in cancer cell survival and in treatment response. qRT-PCR analysis method used may enable high throughput studies to generate molecular biomarker sets for diagnosis and predicting cancer treatment response.

## Introduction

Cancer biomarkers are sought to help in definitive diagnosis, treatment responses, and predicting survival of cancer patients [1], [2]. In order to predict cancer treatment response, reliable markers of cancer cell death and survival pathways and how they are affected by cancer treatment modalities are needed. Available methods to identify such markers include genomics, proteomics and tissue based immunohistochemical staining [3]. Quantitation of cancer biomarker transcripts using real-time quantitative polymerase chain reaction (qRT-PCR) of large samples may help in the search for clinically useful cancer biomarkers that can be integrated into clinical trial design [4].

The desired end-point in the treatment of human cancers is to produce total cancer cell death [5], [6]. Cancer cell death can proceed via apotosis (type I), necrosis or autophagy (type II) [7], [8]. Necroptosis (necrosis) is a programmed cell death pathway that requires the formation of necrosome, has a natural inhibitor (necrostatin1), and requires the functioning complex of RIP1 and RIP3 (serine/threonine kinase receptor interacting proteins 1/3) [9], [10], [11], [12]; necroptosis can be induced by other pathways such as toll-like receptors (TLR), Jun kinases, CD40, SPP1 and glutathione metabolism [13]. Necroptosis and apoptosis have some common regulators. RIP3 can move cells from apoptosis to necroptosis [14]. Induction of autophagy can complement drug-induced cancer cell death [15]. There is growing consensus that all three programmed cell-death pathways are interconnected [16], [17]. Furthermore, one principal feature of most cancers is to resist cell death [18]. Many factors and signaling pathways lead to cancer cell death [8]. Inhibiting autophagy may promote necrotic cell death [19], apoptosis [20], and apoptosis can switch to autophagy [21] and vice-versa [22].

Galectin-3, a member of the galectin family (galectins 1–15), has both anti- and pro-apoptotic effects, expressed in the nucleus and cytoplasm [23], [24], affects Ras-signaling in cancers[25] and nuclear localization may induce resistance to treatment [26], [27]. Galectin-3 is present in many normal tissues and cell types [28] including endothelial cells [29]. Galectin-3 expression has been linked to progression, metastasis and survival of patients with many human cancer types such as breast and thyroid cancers [24], [30], [31]. Galectin-3 regulates other signaling pathways [32]. Galectin-3 signaling has not been linked to autophagy but has BH1 domain of Bcl-2 and interacts with Bcl-2 [33], [34] and thus could interact with the autophagy pathway.

Beclin1 (also known as Atg6) is an autophagy related protein and haploinsufficient tumor suppressor gene and oncogene [35] and is highly expressed in many human cancers. Beclin1 over-expression may promote cancer cell survival [5]. Beclin1 is deleted in some cancers [36]. Beclin1 is a class III phosphatidylinositol 3-kinase [37], [38]. Autophagy genes are regulated by cell-cycle genes (E2-F1) [39], oncogenes [40], [41], [42] and inositol triphosphate receptor [43].

Apoptosis and autophagy are activated by similar signals such as stress, drugs, radiation and are regulated by similar pathways such as Bcl-2, Bax, phosphatase and tensin homologue deleted in chromosome 10 (PTEN), mammalian target of rapamycin (mTOR), Akt, and p53 [5], [8], [16], [20], [44], [45], [46], [47], [48]. This suggests that expressions of galectin-3 may regulate or be related to autophagy in cancers and act as additional mode of influencing cancer progression.

The aim of the study was to determine expression levels of galectin-3 and Beclin1 in both normal and cancer tissues and correlate expression patterns in cancer types.

The present study used qRT-PCR to determine mRNA levels of galectin-3 and Beclin1 in human cancers. Galectin-3 mRNA levels were higher than Beclin1 in all tissue types and over-and under-expression of both galectin-3 and Beclin1 were seen in normal and cancer tissues. The findings support the known effects of galectin-3 on the behavior and progression of certain human cancer types. Beclin1 expression levels suggested contribution to cancer behavior and proposed role in cancer treatment response. The method of qRT-PCR data analysis will be of help in large-scale studies to determine useful cancer cell death biomarkers.

## Materials and Methods

### TissueScan cDNA Panel for Quantitative Real –Time Polymerase Chain Reaction (qRT-PCR)

Origene Oncology TissueScan cDNA panel of 96 samples of human normal (3) and cancer tissues (9)(breast, colon, kidney, liver, ovary, prostate, lung and thyroid) was obtained from Origene Inc (CSRT101; 2–96well pack). Breast tissues were from females only, ages 42–63years. Colon samples were from males and females, ages 42–91years. Kidney samples were from males and females, ages 32–57 years. Lung samples were from males and females, ages 49–79 years. Liver samples were from males and females, ages 21–86 years. Thyroid samples were from females and males, ages 15–74 years. Ovary samples were from females only, ages 31–80years. Prostate samples were males only, ages 53–71years.

### Primers for QRT-PCR

The primers for Galectin-3 (LGALS3 chromosome 14q21–q22, NCBI GenBank ID NM-002306), Beclin1 (BECN1, Chromosome17q21: NCBI GenBank ID 003766) and β-Actin (ACTB; NCBI GenBank ID 001101) are in Table 1 including amplicon size and lengths of primers. The primers were obtained from PrimerBank [49]. The purified oligonucletides were purchased from IDT (Integrated DNA Technologies Inc).

**Table 1:** Primers for Galectin-3, Beclin1 and β-Actin used for qRT-PCR.

Gene Name/ID	Forward 5′-3′	Reverse 3′-5′
LGALS3/NM002306	**TTTTCGCTCCATGATGCGTTA**	**GCCTGTCCAGGATAAGCCC**
BECN1/NM003766	**ACCGTGTCACCATCCAGGAA**	**GAAGCTGTTGGCACTTTCTGT**
ACTB/NM001101	**CATGTACGTTGCTATCCAGGC**	**CTCCTTAATGTCACGCACGAT**

LGALS3 amplicon size = 161, location 5′-3′ = 13-33, 3′-5′ = 173-155. BECN1 amplicon size = 188, location 5′-3′ = 104-123, 3′-5′ = 291-271. ACTB amplicon size = 250, location 5′-3′ = 393-413, 3′-5′ = 642-622.

### Quantitative RT-PCR (qRT-PCR)

Quantitative real-time polymerase chain reactions (qRT-PCRs) were carried out at the Integrated Biomolecular Design Laboratories (IBD) in the Department of Biochemistry, Faculty of Medicine and Dentistry University of Alberta using Applied Biosystems 7900HT Fast Real-Time PCR system (Applied Biosystems Inc CA, USA). The reaction mix contained 5 µL of sample mixed with 15microL of PCR cocktail (primer 2,8 µM and Jump2X ( dNTP 160 µL, ROX 400 µL, SYBER GREEN 1/00 DMSO 50 µL, Jumpstart Taq 240 µL Total 10 mL) at a ratio of 1∶2).Each well was run in duplicate for test primer and reference (β-actin) and a duplicate sample set was run for each primer of interest. The reaction for each well was carried out as follows: 95°C for 3 minutes, followed by 95°C for 15 seconds, 55°C for 15 seconds, 72°C for 1 minute X 40 cycles, then followed by 95°C for 15 seconds, then 60°C for 15 seconds and finally 95°C for 15 seconds. The ABI7900HT software (SD 2.0) was used to obtain raw fluorescence data (Rn and DRn) for analysis.

### qRT-PCR Data Analysis

All raw fluorescence data (Rn) for each well (in duplicate) were uploaded to the Miner website (www.ewindup.info/miner/verson2) for analysis using 4- and 3-parameter logistic regression model to calculate efficiencies (E) and cycle threshold (Ct) values derived from the second derivative maximum of the model [50]; the kinetic model does not require use of standard curve, defines S shaped curve for each PCR, identifies the exponential phase(EP) of the PCR reaction, estimates efficiency (E) using iterative non-linear regression followed by weighted average to fit the appropriate curve of the raw data and Ct derived from second derivative maximum. Miner software provided the results for each well and the average Ct and Efficiency for each well. Samples were in duplicate and the mean/average was used in calculations of efficiency (E) and Ct. Similar methods have been used by others such as the sigmoidal curve fitting model [51], [52], [53] and linear regression model [54]. Many aspects of MIQE guidelines were taken into consideration for methods and analysis [55].

### Statistical Analysis

The statistical software package R (www.r-project.org) was used to calculate relative concentrations (fold change) of mRNA levels of galectin-3 and Beclin1 in the samples using the Miner formula 1/(1+E)∧CT. Gretl software (v1.8.0) was used for plotting and statistical analysis.

## Results

### Galectin3 mRNA Up-regulation in Human Cancer Tissues

**Figure 1:**
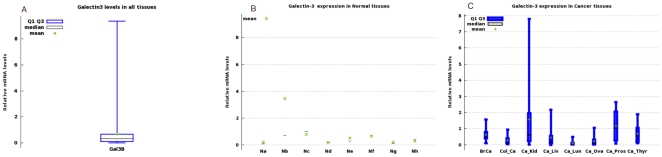
Galectin-3 mRNA levels in Human Normal and Cancer Tissues. (**A**) Box-plot of the overall Galectin-3 expression in all tissue types. (**B**) Box plots of galectin-3 distribution of relative mRNA levels in normal Tissues (Na = normal breast, Nb = Normal colon, Nc = Normal kidney, Nd = Normal liver, Ne = Normal lung, Nf = Normal ovary, Ng = Normal Prostate, Nh = Normal thyroid). (**C**) Box plots of relative mRNA Levels of galectin3 in cancers tissues. BrCa = Breast cancer, Col_Ca = colon cancer, Ca_Kid = Kidney cancer, Ca_Liv = liver cancer, Ca_Lun = lung cancer, Ca_Ova = ovarian cancer, Ca_Pros = Prostate cancer, Ca_Thyr = thyroid cancer (see Table 2a, and b for values for each group).

Overall galectin-3 mRNA levels in all 96 samples is shown in **Fig. 1a**. Minimum value was 1.2e-03 and maximum value was 937e-02 (mean = 67.6047e-02+/−130.3e-02). Relative mRNA levels of galectin-3 in normal tissues are in **Fig. 1b** and for cancer tissues in **Fig. 1c**. The highest level of galectin-3 in normal tissues was in colonic mucosa. Normal and cancer tissues from the liver and lung had the lowest expressions of galectin-3. High galectin-3 expressing cancer tissues were breast, prostate, kidney and thyroid when compared to normal tissues (Tables 2,and 3).

**Table 2:** Summary of normalized galectin-3 mRNA levels in Normal and Cancer Tissues.

Normal Tissue Type	Mean	Cancer Tissue Type	Mean
Normal Breast	21.476e-02	Breast Cancer	61.299e-02
Normal Colon	34.615e-02	Colon Cancer	27.471e-02
Normal Kidney	77.705e-02	Kidney Cancer	156.67e-02
Normal Liver	18.151e-02	Liver Cancer	44.6160e-02
Normal Lung	52.249e-02	Lung Cancer	11.1460e-02
Normal Ovary	66.595e-02	Ovarian Cancer	24.254e-02
Normal Prostate	21.634e-02	Prostate Cancer	116.12e-02
Normal Thyroid	30.185e-02	Thyroid Cancer	68.058e-02

**Table 3:** Summary of minimum, median, and maximum values of relative galectin-3 mRNA levels in normal and cancer tissues to complement Figures 1a and b.

Tissue Type	Minimum	Median	Maximum
Normal Breast	0.01506	0.0764	0.553
Breast cancer	0.102	0.50	1.569
Normal colon	0.318	0.696	9.3714
Colon cancer	0.0337	0.1524	0.9418
Normal kidney	0.1347	0.9965	1.2
Kidney cancer	0.0118	0.6298	7.7939
Normal liver	0.1363	0.20122	0.20705
Liver cancer	0.0012	0.17409	2.1651
Normal lung	0.2548	0.2929	1.0197
Lung cancer	0.0052	0.0588	0.4839
Normal ovary	0.3582	0.7185	0.92118
Ovary cancer	0.01455	0.10754	1.0375
Normal prostate	0.09868	0.10115	0.44919
Prostate cancer	0.07828	1.0284	2.6502
Normal thyroid	0.0213	0.40815	0.47609
Thyroid cancer	0.11338	0.54332	1.8976

### Beclin1/Atg6 mRNA in Human Normal and Cancer Tissues

**Figure 2:**
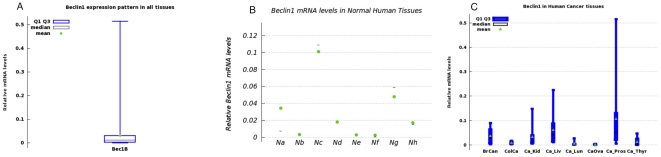
Beclin1/Atg6 relative mRNA levels in Human Normal and Cancer Tissues. (**A**). Box plot of the overall expression in all tissue types. (**B**) Box plots of Beclin1 distribution of mRNA levels in normal tissues (Na = normal breast, Nb = Normal colon, Nc = Normal kidney, Nd = Normal liver, Ne = Normal lung, Nf = Normal ovary, Ng = Normal Prostate, Nh = Normal thyroid). (**C**) Box-whisker plots of the relative Beclin1 mRNA levels in cancer tissues. BrCa = Breast cancer, Col_Ca = colon cancer, Ca_Kid = Kidney cancer, Ca_Liv = liver cancer, Ca_Lun = lung cancer, Ca_Ova = ovarian cancer, Ca_Pros = Prostate cancer, Ca_Thyr = thyroid cancer (see Table 3a and b for values).

Relative mRNA levels for Beclin1, in all 96 tissue types were lower compared to galectin-3 (**Fig. 2a**). Minimum value was 1.27e-05 and maximum value was 5.15e-01 (mean = 3.18056e-02+/−6.4003e-02). The distribution of Beclin1 mRNA in normal tissues is presented in **Fig. 2b** and for cancer tissue types is shown in **Fig. 2c** and Tables 4 and 5. Colon, prostate, and liver cancer tissues had high relative mRNA values.

**Table 4:** Beclin1/Atg6 relative mRNA Levels in Normal and Cancer Tissues.

Normal Tissue Type	Mean	Cancer Tissues	Mean
Normal Breast	3.4437e-02	Breast Cancer	3.598e-02
Normal Colon	.301320e-02	Colon Cancer	.871590e-02
Normal Kidney	10.093e-02	Kidney Cancer	3.1876e-02
Normal Liver	1.77110e-02	Liver Cancer	6.1218e-02
Normal Lung	.254410e-02	Lung Cancer	.536290e-02
Normal Ovary	.242314e-02	Ovary Cancer	.1731e-02
Normal Prostate	4.7847e-02	Prostate Cancer	10.5990e-02
Normal Thyroid	1.68790e-02	Thyroid Cancer	1.30417e-02

**Table 5:** Summary of minimum, median, and maximum values of Beclin1 mRNA levels in normal and cancer tissues to complement Figs. 2a and b.

Tissue Type	Minimum	Median	Maximum
Normal Breast	0.0048	0.00734	0.09116
Breast cancer	0.00241	0.02845	0.08946
Normal colon	0.00102	0.002030	0.005988
Colon cancer	0.0022988	0.00666	0.0169
Normal kidney	0.06459	0.10863	0.12958
Kidney cancer	0.00570	0.011725	0.14817
Normal liver	0.00344	0.0185558	0.03113
Liver cancer	0.011579	0.038995	0.22463
Normal lung	0.0013938	0.0015910	0.004647
Lung cancer	0.0011614	0.002225	0.02738
Normal ovary	0.803e-05	0.000404	0.006816
Ovary cancer	0.2665e-05	0.0018499	0.0037392
Normal prostate	0.018351	0.058766	0.066426
Prostate cancer	0.005257	0.051456	0.51532
Normal thyroid	0.004472	0.014862	0.031303
Thyroid cancer	0.1993e-05	0.0019467	0.04676

### Imbalance of Galectin-3 and Beclin1/Atg6 Expression In Normal and Cancer tissues

**Figure 3:**
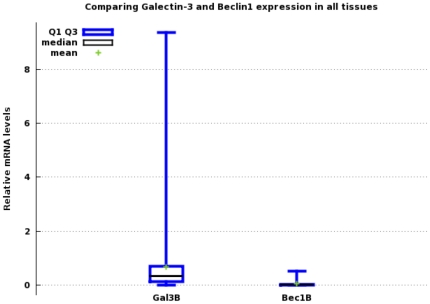
Box plots comparing galectin-3 and Beclin1 mRNA levels in all tissue types. (mean+/−standard deviation) Gal3 = 67.605e-02+/−1.3030 Beclin1 = 3.1806e-02+/−6.4003e-02.

**Figure 4:**
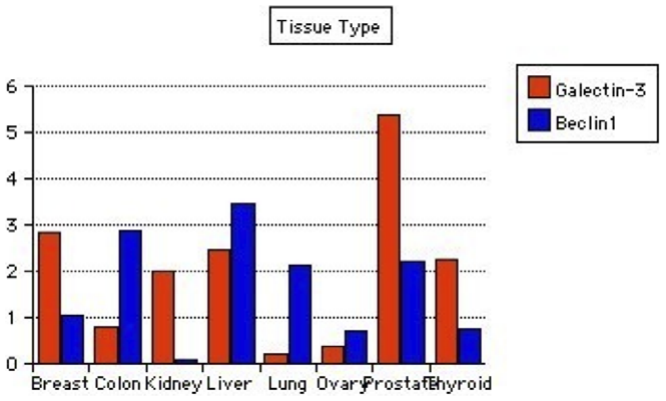
Bar plot using the cancer/normal tissue ratios of galectin-3 and Beclin1 expression from **Tables 2** and **3**. Comparative changes in galectin-3 and Beclin1 expression in individual cancer types are shown.

Expression levels of mRNAs for galectin-3 and Beclin1 in all 96 tissues are shown in **Fig. 3**. Wilcoxon Signed-Rank tests for the differences between galectin-3 and Beclin1 showed p-value of 2.22045e-1(two-tailed). In breast cancer tissues, galectin-3 but not Beclin1 was highly expressed in cancer compared to normal tissue. In colonic cancer tissues, Beclin1 was higher in cancer compared to normal, while galectin-3 was lower compared to normal tissues. Beclin1 level was higher in cancer than normal liver tissues while galectin-3 was low though higher in cancer than normal tissues. Kidney and thyroid cancer tissues had high galectin-3 but lower Beclin1 in cancer compared to normal. In the ovaries, both galectin-3 and Beclin1 levels were lower in cancers compared to normal ovarian tissues. Lung tissues had lower galectin-3 and higher Beclin1 in cancer compared to normal. Prostate cancer tissues had both galectin-3 and Beclin1 levels above normal tissue levels (Tables 2, 3, 4 and 5; Figure 4).

## Discussion

This study was to quantify transcript levels of two cell death markers in human normal and cancer tissues, compare levels between normal and cancer tissues, and compare and correlate levels in the different cancer types. The study found elevated levels of galectin-3 mRNA and relatively low levels of Beclin1 mRNA. The distribution of galectin-3 mRNA levels in normal and cancer tissues are higher than for Beclin1 (Tables 2, 3, 4, and 5); there is no indication that this implies that galectin-3's function in apoptosis interferes with Beclin1's autophagy functions. That interaction, if identified, could impact cancer treatment response. Both galectin-3 and Beclin1 can transport to nuclei [56], [57], reach the Golgi organelle [58] and interact with Bcl-2 [33] and are inhibited by Bcl-2 [44], [59]. Beclin1 is also a therapeutic target [60] as autophagy can increase resistance to irradiation; inhibition of Beclin1 leads to increased sensitivity to irradiation [19].

### Galectin-3 Role in Human in Cancer

Recent observations in a cancer xenograft model indicates that inhibition of galectin-3, using synthetic lactulosyl-1-leucine combined with taxol, reduced lung metastases and increased metastases-free animals [61]; a finding that points to increasing relevance of galectin-3 activities in human cancer. Another study used small molecule inhibition of galectin-3 in thyroid cancer cell lines and showed that inhibition induces apoptosis of the cancer cells and sensitivity to doxorubicin [62]. Cancer stroma angiogenesis may be promoted through matrix metalloproteinase cleavage of galectin-3 N-terminus [63] in both in-vitro angiogenesis models and human cancer tissues. Furthermore, use of galectin-3 inhibitors in-vitro and gal3 (−/−) animals indicate that galectin-3 binds intergrin α5 β3 and induces angiogenesis via vascular endothelial growth factor (VEGF) and b-fibroblast growth factor (bFGF) [64]. In the cancers with elevated galectin-3 and Beclin1 (liver and prostate cancers), targeting both may be of benefit.

In the present study, galectin-3 mRNA levels were highest in breast, lung, kidney, and thyroid cancers when compared to normal tissues. High expressions of galectin-3 in some human cancers indicate progression and metastatic activity of cancer, i.e breast [65], [66], kidney [67], and thyroid [68]. Galectin-3 is a chemoattractant for monocytes and macrophages [69] and expressed in tumor vascular endothelial cells and could function in the feed-back of stroma and cancer cells [70]. Galectin-3 has effects on K-ras signaling in breast cancer cells [25] and its nuclear translocation increases resistance to chemotherapy [71]. Galectin-3 promotes metastasis in experimental breast cancer metastasis models [72]. Galectin-3 is elevated in thyroid cancers with mutated p53 especially poorly differentiated and anaplastic subtypes [73]. High levels of galectin-3 in these subtypes have spurred the use of galectin-3 in imaging of these cancers [68] and as therapeutic target [31]. As was found in this study, galectin-3 is expressed in normal thyroid [74] and the higher levels in thyroid cancers, as seen in the present study, may be used to separate benign and malignant thyroid tissues provided appropriate evidence-based data are taken into consideration [75]. Combining small molecule inhibition of galectin-3 and doxorubicin treatment of thyroid papillary cancer and cell lines increased drug response and apoptosis [62]. In colonic cancers, nuclear localization of galectin-3 is associated with resistance to 5-fluorouracil (5-FU) treatment [76]. In the present study, colon cancers as a group had low galectin-3 levels, though its contribution to progression is unknown. In hepatocellular carcinomas, elevated serum or nuclear galectin-3 indicated histologic grade and vascular invasion [77]; in this study both galectin-3 and Beclin1 are elevated in liver cancer when compared to normal tissues and possibly supports the relative resistance to chemo-radiotherapy. As shown in other studies, galectin-3 was highly expressed in the renal cancers [67]. High levels of galectin-3 in renal cell carcinomas was found in primary and metastatic renal cell carcinomas; metastatic carcinomas had higher levels than primary carcinomas suggesting a role in progression [78]. High galectin-3 levels in prostate cancers in this study may support the findings in model animal studies. In the prostate cancer cell line LNCaP that lacks galectin-3 (due to promoter hypermethylation), induced expression of galectin-3 leads to reduced drug-induced apoptosis [71]. Similarly, over-expression of galectin-3 in the prostate cancer cell line LNCaP that lacks galectin-3 expression led to inhibition of tumor growth [79]. Galectin-3 may be cleaved by proteases during prostate cancer progression, and PC3 prostate cancer cell line with reduced galectin-3 showed reduced tumor growth in xenografts [80]. High levels of galectin-3 in the prostate cancer samples could indicate, low stage and less progressive disease [81], [82].

### Beclin1's Input in Cancer Behavior

In contrast to the galectin-3 findings, Beclin1 was generally down regulated in all tissue types and in many cancer subtypes. In hepatocellular carcinomas, Beclin1 was increased compared to normal and reduction in Beclin1 was reported as predictor of disease free survival and aggressive cancers had low levels of Beclin1 [83]; the findings in liver cancer could suggest less aggressive cancers. Beclin1 was found to regulate estradiol activity in breast cancer [84] and relatively normal levels found may indicate estrogen activity. Beclin1 levels in prostate cancer may support the findings in model animal studies. Several studies have shown that treatment of prostate cancer cells with sulforaphane [85] and inhibition of mTOR ( target of rapamycin) induces autophagy and increased response to irradiation [86]. Prostate cancers had Beclin1 higher than normal suggesting possible radiation response. There are no direct studies of changes in Beclin1 in renal cancer, though small molecule inhibition of VHL (von Hippel Landau) in cancer cells induced autophagy and reduced growth [87]. In this study, renal cancers expressed Beclin1 below normal tissues and may suggest lack of activation of autophagic cell death. In ovarian cancers, induction of autophagy leads to tumor dormancy [88]. In this study, ovarian cancers had Beclin1 levels below that in normal tissues indicating absence of dormancy, and possibly aggressive behavior. Expression levels of Beclin1 in ovarian, prostate and breast cancers may be influenced by known deletions that occur in these cancers [36].

### Galectin-3 and Beclin1 Together in Human Cancers

Deciphering the relevant cell death pathways in human cancer may provide a way to select specific treatments to avoid unwanted responses. Regulations of apoptosis and necroptosis do overlap but are also unique to each cell death model [9], [10]. Therefore, suppression of galectin-3 and its pro-apoptosis functions will promote necroptosis via RIP1 and RIP3 [11], [14]. Proposed models suggest that when autophagy is intact and functioning, cells will undergo apoptosis when apoptosis pathway is preserved and go to survival if apoptosis is deranged; on the other side, if autophagy is defective but apoptosis is intact, cells will undergo apoptosis but survive if apoptosis is deficient [89]. In human cancers with high galectin-3 (presumed pro-apoptosis) and Beclin1, use of small molecule inhibition of galectin-3 to enhance treatment response may be beneficial. A recent study describes the use of siRNA and GSC-100/MCP (modified pectin citrate) to inhibit galectin-3 in prostate cancer cell of PC3 and enhance cisplatin treatment response [90]. The present study and others suggest the relevance of predetermining the presence of cell death pathway markers in cancers to optimize use of combined drug and small molecule inhibitors.

### Study Limitations

The present study explored the use of qRT-PCR in determining expression levels of two cancer cell death markers. Correlative proteomics will benefit such studies to define correlating protein content and or localization and provide additional support for transcript levels. In prostate cancer and cell line LNCaP, promoter methylation status affected tissue expression of galectin-3; hence proteomics may need correlation with methylation status [91]. Survival and treatment responses are not part of the present study as post-treatment follow-up and survival data were not available. Correlating with survival and treatment response will be essential components of larger samples and studies (normal and cancer greater than 9 samples) aiming to define benefits of predefined cancer cell death biomarkers for clinical use. Emerging methods of next generation sequencing may need validation with enhanced methods of qRT-PCR.

### Utility of Kinetic/Regression Models for qRT-PCR

The present study also explores the use of Miner software (2.0) for analysis of all quantitative polymerase-chain reaction data [50]. This and other methods reported by other investigators do not require the use of standard curve and use linear or non-linear regression modeling [51], [52], [53], [54], [92] and could enhance the ability to conduct high-throughput quantitative real-time polymerase chain reaction(qRT-PCR) studies for corroborating, and validating other microarray experiments.

### Conclusions

The roles of galectin-3 and Beclin1 in apoptosis and autophagy are well described. Galectin-3 and Beclin1 roles in cancer progression and treatment response are supported by the expression patterns in this study. The study used raw fluorescence qRT-PCR data and Miner software (Miner 2.0) that uses logistic regression model to calculate individual well efficiency and Ct values. The method of qRT-PCR analysis lends itself to using large numbers of tissue sets and may be useful in creating biomarker sets based on gene networks that can be used for diagnosis and predicting cancer treatment response.
